# Molecular Patterns of Neurodevelopmental Preconditioning: A Study of the Effects of Antenatal Steroid Therapy in a Protein-Restriction Mouse Model

**DOI:** 10.1155/2014/193816

**Published:** 2014-03-13

**Authors:** Clarissa Velayo, Takuya Ito, Yupeng Dong, Miyuki Endo, Rika Sugibayashi, Kiyoe Funamoto, Keita Iida, Nobuo Yaegashi, Yoshitaka Kimura

**Affiliations:** ^1^Department of Obstetrics & Gynecology, Tohoku University Graduate School of Medicine, 1-1 Seiryomachi, Aoba-ku, Sendai, Miyagi 980-8574, Japan; ^2^International Advanced Research and Education Organization, Tohoku University, Sendai, Miyagi 980-8574, Japan

## Abstract

*Introduction*. Prenatal programming secondary to maternal protein restriction renders an inherent susceptibility to neural compromise in neonates and any addition of glucocorticosteroids results in further damage. This is an investigation of consequent global gene activity due to effects of antenatal steroid therapy on a protein restriction mouse model. *Methods*. C57BL/6N pregnant mice were administered control or protein restricted diets and subjected to either 100 **μ**g/Kg of dexamethasone sodium phosphate with normosaline or normosaline alone during late gestation (E10–E17). Nontreatment groups were also included. Brain samples were collected on embryonic day 17 and analyzed by mRNA microarray analysis. *Results*. Microarray analyses presented 332 significantly regulated genes. Overall, neurodevelopmental genes were overrepresented and a subset of 8 genes allowed treatment segregation through the hierarchical clustering method. The addition of stress or steroids greatly affected gene regulation through glucocorticoid receptor and stress signaling pathways. Furthermore, differences between dexamethasone-administered treatments implied a harmful effect during conditions of high stress. Microarray analysis was validated using qPCR. *Conclusion*. The effects of antenatal steroid therapy vary in fetuses according to maternal-fetal factors and environmental stimuli. Defining the key regulatory networks that signal either beneficial or damaging corticosteroid action would result in valuable adjustments to current treatment protocols.

## 1. Introduction

The concept of the fetal genome is no longer that of a static framework inherited from paternal and maternal sources but a malleable scaffold constantly adapting to stimuli. This is most evident in studies involving fetal programming due to the effects of nutritional variation and glucocorticoid exposure [1]. Here, we examined the resulting fetal molecular preconditioning due to antenatal steroid therapy using a protein-restriction mouse model. This model was first designed in a previous study [2] as a novel approach to evaluate postnatal adaptive responses due to varied prenatal nutritional conditions and the addition of stress or steroids. Molecular evidence revealed that prenatal programming secondary to maternal protein restriction rendered an inherent susceptibility to neural compromise in neonates and any further addition of antenatal steroids may be detrimental to these already injury-prone offspring. Thus, an examination of underlying molecular mechanisms in the fetus was warranted to elucidate the effects seen postnatally.

Understanding any subtle changes in the fetus induced by these factors and their correlation with phenotypic outcomes in the adult would facilitate early detection of either well-being or disease. Current biomolecular techniques such as microarray analysis have allowed the investigation of global gene expression and subsequently, the parallel data mining of gene transcripts of interest as well as the discovery of new gene involvement. Moreover, gene expression profiles through clustering of significant genes have shown promising potential as diagnostic panels. All these have led to the rapid identification of biomarkers for disease conditions and their associated regulatory pathways [3]. Using these advancements, a panoramic view of genetic movement in utero is presented.

## 2. Materials and Methods

### 2.1. Experimental Animals

Female C57BL/6N mice about 6 weeks old provided by the Institute for Animal Experimentation, Tohoku University Graduate School of Medicine, were maintained under controlled lighting (12-hour light cycles) and temperature (24°C). These were allowed for free access to food (AIN-93G: Oriental Yeast Co., Ltd., Tokyo, Japan) and water during a 2-week acclimatization period after which each female was time mated with a male.

### 2.2. Treatment Groups

Pregnant females (*n* = 36) were then housed singly and administered either control (C) or protein restricted (PR) diets ad libitum all throughout pregnancy (Embryonic stage, E0 to E17). These were then further subdivided into 6 groups and subjected to either plain normosaline solution (C-S, PR-S) or 100 *μ*g/Kg dexamethasone sodium phosphate (Decadron, MSD K.K., Tokyo, Japan) in normosaline solution (C-D/S, PR-D/S) by subcutaneous injection daily during late gestation (E10 to E17). Nontreatment groups were also included (C, PR). All injections were performed between 12 nn and 2 pm. Maternal weights on days E0, E10, and E17 were recorded, as well as fetal weights on E17. On embryonic day 17, whole brain samples collected from 2 male and 2 female fetuses from each litter were supercooled in liquid nitrogen and stored at −80°C.

### 2.3. DNA Chip Analysis

A total of 6 Toray 3D-Gene Mouse Oligo chip 24 K (Toray Industries, Inc., Tokyo, Japan) microarrays were analyzed per treatment. Each chip utilized a 0.5 *μ*g portion of combined total RNA from a matched pair of male and female samples. RNA was amplified and labeled using an Amino Allyl MessageAmp II aRNA Amplification kit (Life Technologies Japan Ltd.) according to the manufacturer's instructions. Each sample of aRNA was labeled with fluorescence Cy3 or Cy5 and cohybridized at 37°C for 16 hours. These were subsequently washed and dried. Hybridization signals were scanned using Scan Array Express (Perkin Elmer, MA, USA) and global background analysis was performed using GenePx Pro (MDS Analytical Technologies, CA, USA). All 36 arrays were then normalized together as one experiment to reduce nonbiological variability.

### 2.4. Quantitative PCR (qPCR)

To validate microarray results, qPCR was performed on 2 selected genes, microtubule-associated protein 1b (*Mtap1b*) and 3-hydroxy-3-methylglutaryl-CoA synthase 1 (*Hmgcs1*), using the C and C-D/S treatments. Total RNA was extracted from whole fetal brains (*n* = 6 per treatment) using QIAzol Lysis Reagent (QIAGEN, Hilden, Germany) and cleaned with an AllPrep DNA/RNA Mini kit (QIAGEN, Hilden, Germany) according to the manufacturer's protocol. Complimentary DNA was synthesized using the Superscript III First-Strand Synthesis System (Invitrogen, Carlsbad, CA) and quantitative PCR was conducted with EXPRESS SYBR GreenER Supermix with Premixed ROX (Invitrogen, Carlsbad, CA) on an Eppendorf* Realplex* ^*2*^ Mastercycler (Eppendorf, Hamburg, Germany). Amplified transcripts were quantified and normalized against hypoxanthine phosphoribosyltransferase 1 (*Hprt1*). Primer sequences of the selected genes and housekeeping gene are provided.

### 2.5. Statistical Analysis

Microarray data were subjected to *t*-test analyses with standard Bonferroni correction for multiple comparisons. The *P* value was set at 0.05 and a threshold of 1.5-fold was applied to determine significantly regulated genes. These were subjected to an ontological review and a subset of neurodevelopmental genes for genetic profiling was determined by hierarchical clustering. Targeted gene transcripts of interest on the microarrays were treated to one-way ANOVA with post-hoc analysis (Bonferroni post-test). Confirmation of selected genes by qPCR was validated through Fold Change Analysis (threshold of 1.5-fold). Data management, statistical analysis, and gene ontology were performed using geWorkbench software (https://gforge.nci.nih.gov/frs/?group_id=78) and MGI Gene Ontology Tools (http://www.informatics.jax.org/gotools/).

## 3. Results

### 3.1. Mouse Model

**Figure 1:**
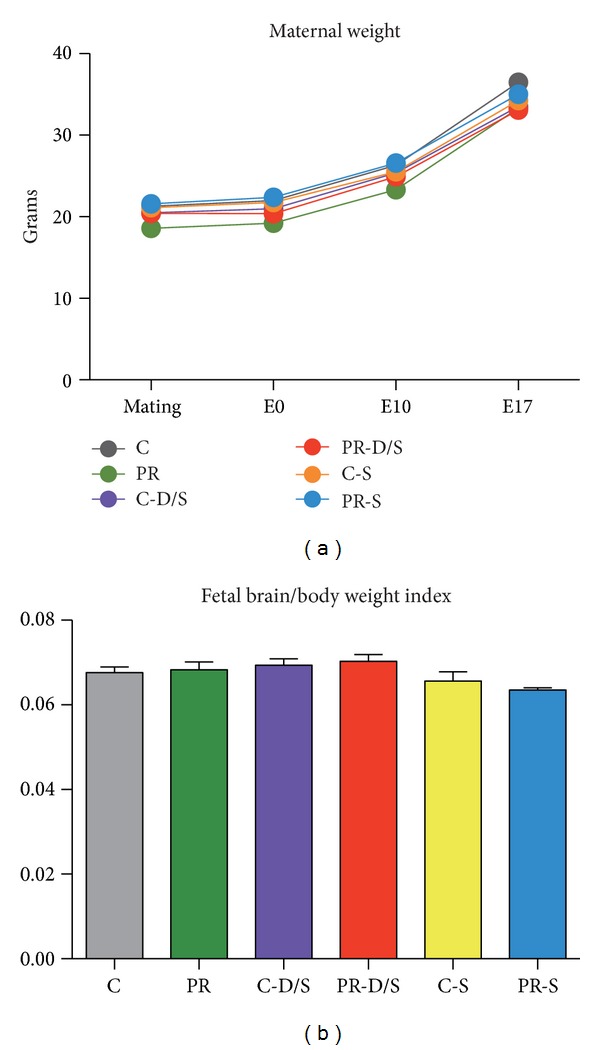
Effects of treatment on animal models. (a) Each data point represents maternal weight as mean ± SEM (*n* = 4 per treatment). Two-way ANOVA indicates a significant treatment effect (*P* < 0.0001) and time effect (*P* < 0.0001). Bonferroni posttest indicates that all treatments were similar to the C group. (b) Mean fetal brain to body weight indices across treatments ± SEM were not significantly different by one-way ANOVA (*n* = 96).

Mean maternal weight gain patterns, between groups, were similar before (E0 to E10) and during treatment (E10 to E17). Mean fetal brain to body weight indices on sampling day E17 were not significantly different (Figure 1).

### 3.2. Global Gene Changes

**Figure 2:**
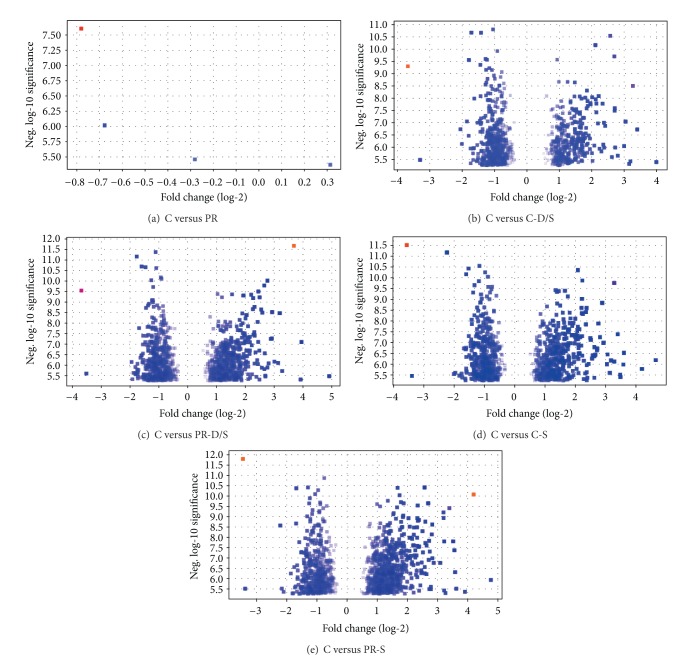
Volcano plots of individual *t*-test analyses using Standard Bonferroni correction and *P* < 0.05 between the control group and all other treatment groups.

**Figure 3:**
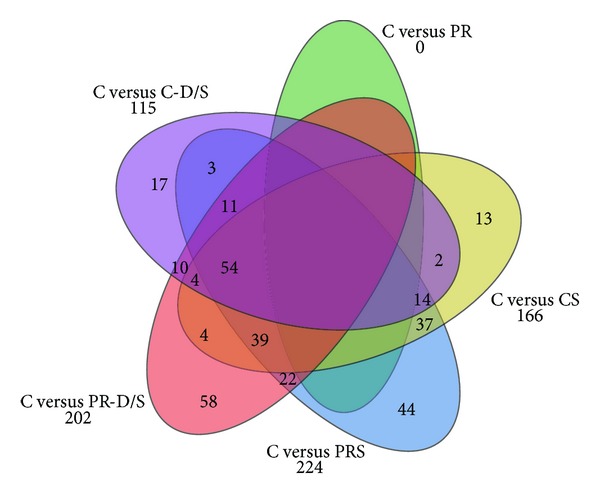
Diagram of 332 significant genes showing areas of overlapping regulation between treatments.

Microarray analyses of 23,522 probe transcripts presented 10,946 genes without absent calls or unreadable hybridization signals. There were more upregulated genes as compared to downregulated genes across all treatment groups (Figure 2, Table 1). The combined number of significant genes regulated from all treatment groups versus the control was 332 (Figure 3). Subsequent gene ontology analysis revealed that ongoing cell organization and biogenesis, developmental processes, and transport were most rampant in the global expression survey. The discovery of genes uniquely activated per treatment and sharing similar ontologies facilitated individual treatment characterization (Table 2(a)). Associated genes were found for protein restriction, cell adhesion genes in both PR-D/S only (*Col1a1*,* Atp1b2*,* Ctnnd1*,* Rpsa*, and* Fat4*), and PR-S only (*Cdh2*,* Edil3*, and* Astn1*); for dexamethasone treatment, stress response genes in both C-D/S only (*Brsk1*,* Rarres2*), and PR-D/S only (*Klk8*,* Myo6*,* Ndufa6*,* Col1a1*,* Mapk8*, and* Phlda3*); and for both protein restriction, dexamethasone treatment, and DNA metabolism genes for PR-D/S only (*Tcf3*,* Mapk8*). Overall, neurodevelopmental genes were overrepresented among those significantly regulated (Table 2(b)) and were associated with* nervous system development* (*Pbx3*,* Eif2b5*,* Nlgn1*,* Mark4*,* Atp2b2*,* Nrxn3*,* Ncam1*,* Tnik*,* Slitrk1*,* Cdh2*,* Synj1*,* Palm*,* Nrp1*,* Rpl24*,* Mtap2*,* Rpgrip1*,* Pou3f2*,* Gabrb3*,* Lrp6*,* Sulf2*,* Ank3*,* Ccdc88a*,* Atrx*,* Nr2c2*,* Opa1*,* Abi2*,* Mtap1b*,* Tcf3*,* Syne1*,* Mapk8*,* Golga2*,* Atxn2*,* Gfra1*,* Snap91*,* Slitrk5*,* Celsr2*,* Emx2*,* Klk8*,* Myo6*,* Scn2a1*,* Sema3c*, and* Kif5c*);* generation of neurons* (*Pbx3*,* Nlgn1*,* Atp2b2*,* Ncam1*,* Tnik*,* Slitrk1*,* Cdh2*,* Synj1*,* Palm*,* Nrp1*,* Rpl24*,* Mtap2*,* Rpgrip1*,* Pou3f2*,* Gabrb3*,* Lrp6*,* Ank3*,* Ccdc88a*,* Abi2*,* Mtap1b*,* Tcf3*,* Syne1*,* Mapk8*,* Golga2*,* Atxn2*,* Gfra1*,* Slitrk5*,* Snap91*,* Celsr2*,* Emx2*,* Klk8*,* Myo6*,* Sema3c*,* Kif5c*,* Robo2*,* Arhgef2*,* Brsk1*,* Pou3f4*, and* Sox11*);* neuron differentiation* (*Pbx3*,* Nlgn1, Atp2b2, Ncam1, Tnik, Slitrk1, Cdh2, Palm, Nrp1, Rpl24, Mtap2, Rpgrip1, Pou3f2, Gabrb3, Lrp6, Ank3, Ccdc88a, Abi2, Mtap1b, Tcf3, Syne1, Mapk8, Golga2, Gfra1, Atxn2, Slitrk5, Snap91, Celsr2, Emx2, Klk8, Myo6, Sema3c, Kif5c, Robo2, Brsk1, Pou3f4, *and* Sox11*);* neurogenesis* (*Pbx3, Eif2b5, Nlgn1, Atp2b2, Ncam1, Tnik, Slitrk1, Cdh2, Synj1, Palm, Nrp1, Rpl24, Mtap2, Rpgrip1, Pou3f2, Gabrb3, Lrp6, Ank3, Ccdc88a, Abi2, Mtap1b, Tcf3, Syne1, Mapk8, Golga2, Atxn2, Gfra1, Slitrk5, Snap91, Celsr2, Emx2, Klk8, Myo6, Sema3c, Kif5c, Robo2, Arhgef2, Brsk1, Pou3f4, *and* Sox11*);* neuron development* (*Pbx3, Abi2, Mtap1b, Syne1, Mapk8, Golga2, Nlgn1, Atxn2, Gfra1, Snap91, Slitrk5, Celsr2, Atp2b2, Ncam1, Tnik, Slitrk1, Klk8, Cdh2, Myo6, Sema3c, Palm, Kif5c, Nrp1, Rpl24, Robo2, Mtap2, Rpgrip1, Brsk1, Pou3f2, Gabrb3, Ank3, *and* Ccdc88a*); and* neuron projection development* (*Abi2, Mtap1b, Syne1, Mapk8, Golga2, Nlgn1, Atxn2, Gfra1, Snap91, Slitrk5, Celsr2, Ncam1, Tnik, Slitrk1, Klk8, Cdh2, Myo6, Sema3c, Palm, Kif5c, Nrp1, Rpl24, Robo2, Mtap2, Brsk1, Pou3f2, Ank3, *and* Ccdc88a*).

### 3.3. Gene Expression Profiling

**Figure 4:**
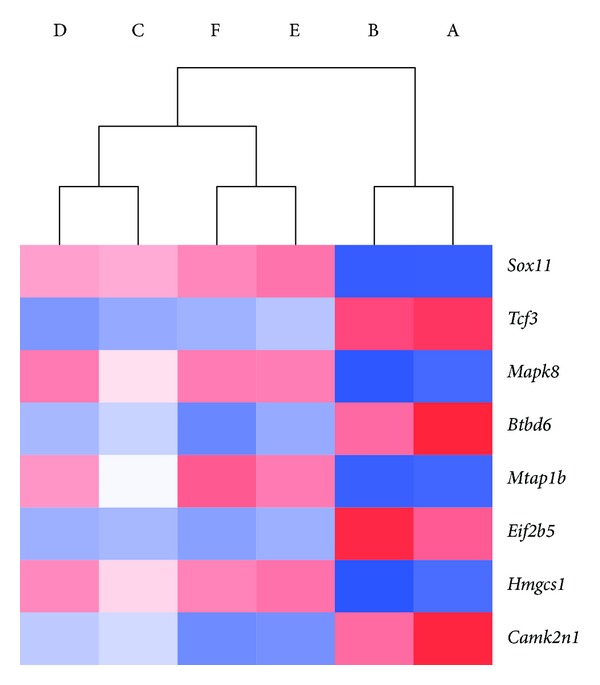
Hierarchical clustering using the average linkage method and Pearson's correlation as the clustering metric resulted in 8 neurodevelopmental genes for genetic profiling: (A) C; (B) PR; (C) C-D/S; (D) PR-D/S; (E) C-S; (F) PR-S.

A subset of 8 genes out of 332 was filtered through the hierarchical clustering method allowing segregation of treatments (Figure 4). An assessment of individual biological themes within the subset revealed neurodevelopmental roles and distinct causal relationships with glucocorticoid treatment and protein restriction (Table 3).

### 3.4. Targeted Genes of Interest

**Figure 5:**
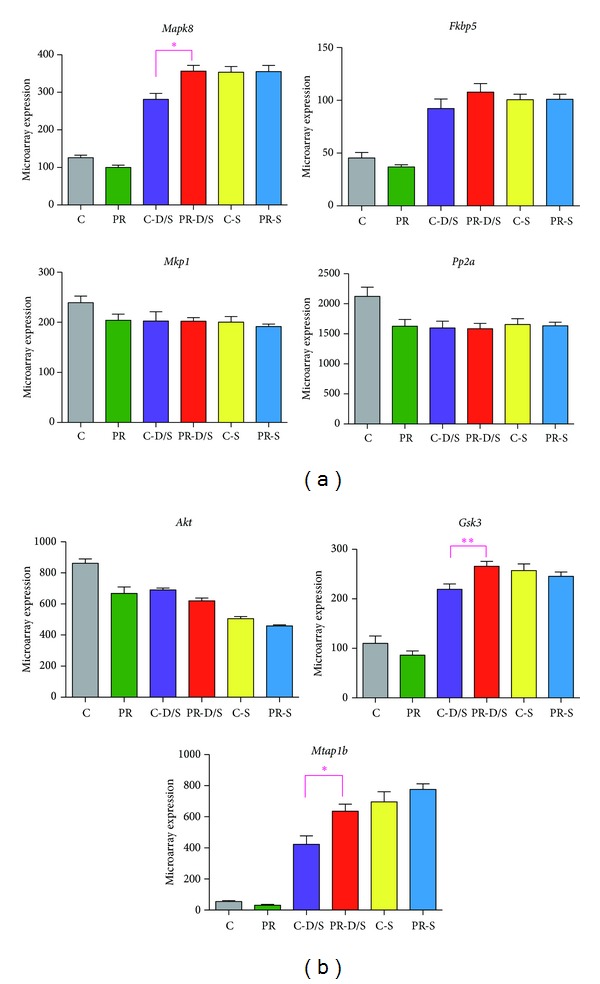
Targeted genes of interest related to stress signaling pathways. (a) One-way ANOVA of gene expression patterns revealing an overall reduction of glucocorticoid receptor (GR) activity included* Mapk8*,* Fkbp5*,* Mkp1*, and* Pp2A*. (b) One-way ANOVA of gene expression patterns for* Akt* and* Gsk3* and* Mtap1b* revealing ongoing neurodysgenesis (*n* = 6 per treatment; mean ± SEM; one-way ANOVA *P* < 0.0001; Bonferroni *P < 0.05 and **P < 0.1).

The addition of stress or steroids (-S and -D/S groups) greatly affected gene regulation leading to further investigation of genes related to glucocorticoid and stress signaling pathways.* Mapk8, Fkbp5, Mkp-1, Pp2A, Akt, *and* Gsk3* exhibited expression patterns across treatment groups that corresponded to an overall reduction in glucocorticoid receptor (GR) activity in the -D/S and -S groups. Most significant of which were the marked differences between C-D/S and PR-D/S (*Mapk8, Gsk3, *and* Mtap1b*) emphasizing the disparate effect on varying nutritional conditions due to dexamethasone administration (Figures 5(a) and 5(b)).

### 3.5. Quantitative PCR Validation

**Figure 6:**
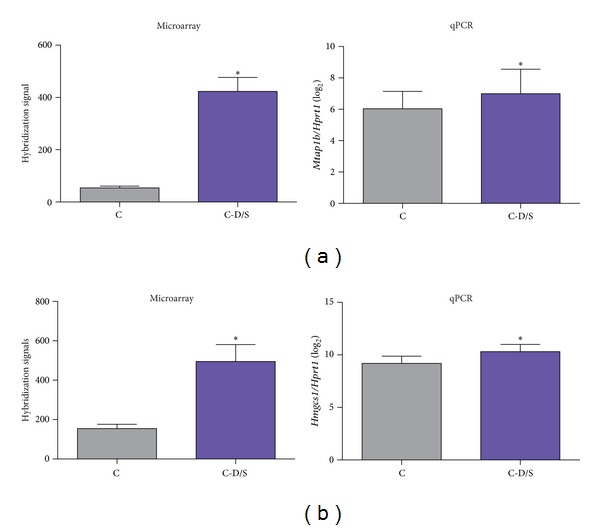
qPCR validation of microarray analysis. Two neurodevelopmental genes,* Mtap1b* and* Hmgcs1*, that were significantly changed between C and C-D/S groups in the microarray analysis (represented as mean hybridization signals + SEM) were compared with qPCR (mean ± SEM transcript signals normalized against* Hprt1*). When comparing non-logged fold changes by the ratio method, there was an overall good agreement between qPCR and microarray analysis with a fold change of at least 1.5* observed. (*n* = 6 per treatment).

qPCR results demonstrated a good agreement with the microarray data for* Mtap1b* and* Hmgcs1* (Figure 6).

## 4. Discussion 

The maternal-fetal compartment serves to cushion the fetus from environmental stimuli, but beyond normal circumstances, prenatal conditioning invariably occurs. Microarray analysis allowed for a panoramic view of gene activity along two levels: through the global expression of genes and through individual treatment groups and their association with one another. In general, ongoing cell organization and biogenesis, developmental processes, and transport were most rampant in the global expression survey (Table 2(a)). The dominance of these particular gene groups is expected in the developing fetus just as genes for growth and maturation are more likely activated in neonates. Regardless, these increased gene frequencies most likely demonstrate consequent fetal reactions to acquired insults from protein restriction and glucocorticoid exposure either as compensatory regulation or protective feedback. They signaled changes within a fetus long previously believed to be immune prior to the conception of the Barker theory [12].

Interestingly, neurodevelopmental genes were overrepresented among the significantly regulated genes. This was exemplified by recurring themes in biological processes related to ongoing brain development during fetal stages: multipotent progenitor differentiation and neuronal migration. Their increased expression over other gene systems emphasized the significance of fetal neuroplasticity even to the detriment of visceral organ growth similar to physiologic brain sparing. This also underlined the early dependence on brain-controlled pathways that trigger bodily functions during stages when less developed organs have not yet attained full functional independence.

Our mouse model simulated conditions of multifactorial environmental impact. The isolation of distinct genes associated with individual factors of protein restriction, dexamethasone, and stress were complimentary to known gene networks.

### 4.1. Protein Restriction Associated with Cell Adhesion Genes

Maternal nutrition plays a critical role in fetal growth and development. Studies of these genes uniquely regulated in both PR-D/S (*Col1a1, Atp1b2, Ctnnd1, Rpsa, *and* Fat4*) and PR-S only (*Cdh2, Edil3, *and* Astn1*) emphasized the crucial role of nutritional factors in maintaining the integrity of cell interaction.* Col1a1*, a known marker of fibrosis and aging, has been linked to alterations in oxidative and antioxidant defense capacity in cells due to poor maternal nutrition [13]. On the other hand, modifications in* Atp1b2, Edil3,* and* Astn1* during development lead to glial dysfunction [14–16]. Moreover, various studies on nutritional factor effects and progenitor cell differentiation included* Ctnnd1, Fat4, *and* Cdh2* [17–19].

### 4.2. Dexamethasone Associated with Stress Response Genes

Fetal dexamethasone exposure impairs development in various cell types eliciting a dose-dependent stress response [20]. In the brain, the pituitary is the site of action of administered dexamethasone in the blockade of stress induced hypothalamic-pituitary axis (HPA) activation. The latter involves the stimulation of brain receptors, primarily, those of glucocorticoid receptors (GR) by both exogenous and endogenous corticosterone. During conditions of stress, the HPA axis releases reactive feedback which suppresses increased excitability allowing recovery from stress induced activation and facilitation of memory storage. The addition of dexamethasone can partially deplete the brain of corticosterone and in turn suppress the fetal HPA axis. Studies that included the genes uniquely regulated in C-D/S (*Brsk1*,* Rarres2*) and PR-D/S (*Klk8*,* Myo6*,* Ndufa6*,* Col1a1*,* Mapk8*, and* Phlda3*) report their important roles in neuroregulation and adaptation to stress responses during brain development [21–25]. Furthermore, expression patterns of targeted genes related to stress signaling pathways revealed decreased GR activity:* Mapk8* (mitogen activated protein kinase 8) and* Fkbp5 *(FK506 binding protein 5), both GR inhibitors [26, 27], were increased in the -D/S and -S groups;* Mkp1* (mitogen activated protein kinase phosphatase 1) and* Pp2a* (protein phosphatase 2), both Map kinase inhibitors [28, 29], were decreased in the -D/S and -S groups (Figure 5(a)).

### 4.3. Convergent Effects of Maternal Nutrition and Dexamethasone Associated with DNA Metabolism

**Figure 7:**
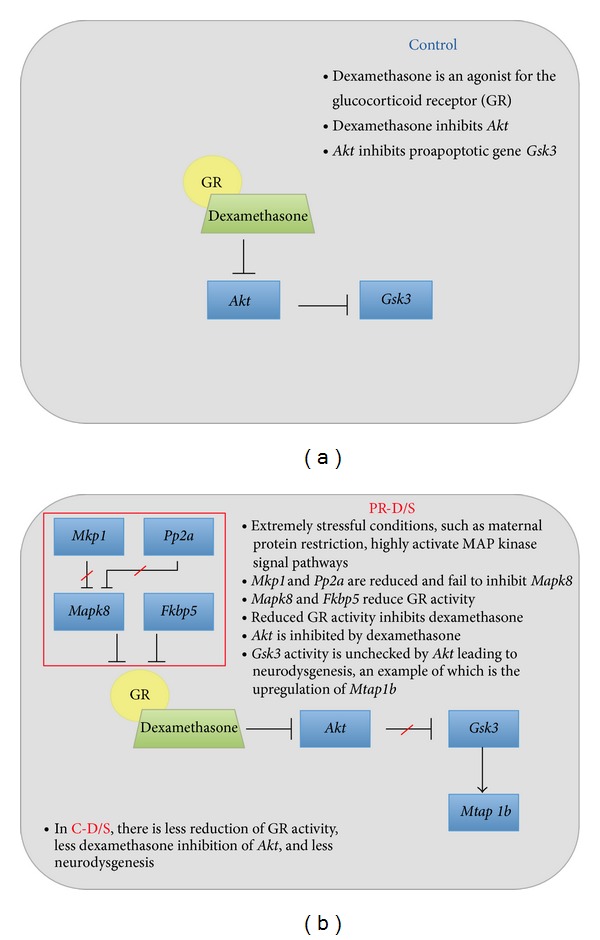
A diagram interrelating the targeted genes of interest with regards to conditions in the control and -D/S groups. (a) Control group and (b) -D/S groups.

Genes associated with PR-D/S only (*Tcf3 *and* Mapk8)* on microarray analysis, as well as two targeted genes (*Akt *and* Gsk3*) all function in metabolic gene networks, especially for glucose metabolism in DNA synthesis. Here, their specific expression patterns between -D/S groups underscored the most significant observation in this study, which was the apparent harmful effect of dexamethasone to fetuses in highly stressed conditions (PR-D/S). A diagram interrelating the targeted genes of interest with regard to GR activity is shown in Figures 7(a) and 7(b). Previous reports have stated that a loss of GR activity reduces dexamethasone inhibition of* Akt* (thymoma viral protooncogene 1), which in turn decreases* Akt* inhibition of* Gsk3 *(glycogen synthase kinase 3), a proapoptotic gene [30–32]. This beneficial effect was seen in the C-D/S group. But in the PR-D/S group, a highly stressed condition, dexamethasone was evidently harmful. This disparate pattern between C-D/S and PR-D/S, significant in* Mapk8, Gsk3, *and* Mtap1b *(*P* < 0.0001), denotes the possibility of ongoing altered neurodevelopment or even neurodysgenesis (Figure 5(b)).

The process of data mining revealed the association between regulated genes unique to individual treatment groups and certain biologic processes. Their correlation provided a better understanding of underlying pathophysiology and a glimpse of key pathways for future focused studies. One possible application is the development of gene panels for genetic expression profiling as diagnostic tools. Hierarchical clustering programs currently allow the generation of gene maps to be capable of distinguishing between phenotypes. In our simulation, highly regulated neurodevelopmental genes were used and these successfully segregated treatments between microarrays (Figure 4).

Our findings strengthen our previous study's assertion that fetal programming secondary to maternal protein restriction renders an inherent susceptibility to neural compromise in offspring and that the addition of dexamethasone to this vulnerable group results in further injury. In future studies, the investigation of both sex-specific and transgenerational effects is necessary as glucocorticoids influence endocrinological pathways differently in males and females. Also, timing of exposure to glucocorticoids as well as dosage studies is no less relevant especially in the light of reported evidence that a single course of therapy profoundly affects the fetal HPA axis [33–35].

In conclusion, the effects of antenatal steroid therapy can vary for each fetus according to maternal-fetal factors and concurrent environmental stimuli. Further elucidating regulatory networks that can mark the turning point between beneficial or damaging corticosteroid actions would result in valuable adjustments of current treatment protocols. The ability to recognize conditions highly vulnerable to damage would also expand the possibility of tailored medicine more suitable to each individual's needs. Current biomolecular techniques are powerful tools in this field of study but further validation between animal and true clinical models is required.

## Figures and Tables

**Table 1:** Summary of significantly regulated genes. Determination of upregulated and downregulated significant genes based on paired *t*-tests with standard Bonferroni correction for multiple comparisons (*P* < 0.05; thresholds of >1.5 for upregulated genes and <−1.5 for downregulated genes).

*t*-test*	Regulated genes	Fold change	
		↑(>1.5)	↓(<−1.5)
C versus PR	4	0	0
C versus C-D/S	751	91	24
C versus PR-D/S	974	158	44
C versus C-S	896	141	25
C versus PR-S	1125	184	40

*P < 0.05.

**Table:** (a) Comparison of gene ontology slim terms of biological processes involved for the total number of genes on the microarray as compared to the combined significantly regulated genes and the genes uniquely regulated per treatment

Biological process	Gene count (frequency)					
	Total number of genes^†^	Significant genes^‡^	C-D/S only	PR-D/S only	C-S only	PR-S only
Cell adhesion	460 (0.04202)	21 (0.06325301)	0 (0.00000)	5 (0.08621)^§^	0 (0.00000)	3 (0.06818)^§^
Cell-cell signaling	403 (0.03682)	22 (0.06626506)	2 (0.11765)	5 (0.08621)	2 (0.15385)	0 (0.00000)
Cell cycle and proliferation	1329 (0.12141)	35 (0.10542169)	0 (0.00000)	6 (0.10345)	2 (0.15385)	0 (0.00000)
Death	946 (0.08642)	29 (0.08734940)	2 (0.11765)	5 (0.08621)	0 (0.00000)	2 (0.04545)
Cell organization and biogenesis	2327 (0.21259)	96 (0.28915663)	6 (0.35294)	14 (0.24138)	4 (0.30769)	11 (0.25000)
Protein metabolism	2199 (0.20090)	61 (0.18373494)	3 (0.17647)	13 (0.22414)	2 (0.15385)	7 (0.15909)
DNA metabolism	438 (0.04001)	20 (0.06024096)	0 (0.00000)	2 (0.03448)^¶^	0 (0.00000)	0 (0.00000)
RNA metabolism	2020 (0.18454)	65 (0.19578313)	7 (0.41176)	14 (0.24138)	3 (0.23077)	7 (0.15909)
Other metabolic processes	1657 (0.15138)	57 (0.17168675)	4 (0.23529)	10 (0.17241)	2 (0.15385)	9 (0.20455)
Stress response	1156 (0.10561)	29 (0.08734940)	2 (0.11765)^ß^	6 (0.10345)^ß^	0 (0.00000)	0 (0.00000)
Transport	1929 (0.17623)	72 (0.21686747)	4 (0.23529)	13 (0.22414)	4 (0.30769)	5 (0.11364)
Developmental processes	2000 (0.18272)	83 (0.25000000)	5 (0.29412)	13 (0.22414)	5 (0.38462)	10 (0.22727)
Signal transduction	2111 (0.19286)	59 (0.17771084)	0 (0.00000)	10 (0.17241)	2 (0.15385)	3 (0.06818)
Unknown biological processes	0 (0.00000)	0 (0.00000000)	0 (0.00000)	0 (0.00000)	0 (0.00000)	0 (0.00000)
Other biological processes	10946 (1.00000)	331 (0.99698795)	16 (0.94118)	57 (0.98276)	12 (0.92308)	43 (0.97727)
All biological processes	10946 (1.00000)	332 (1.00000000)	17 (1.00000)	58 (1.00000)	13 (1.00000)	44 (1.00000)

^†^Only genes without any absent calls were included in the analysis from 23,522 transcripts on the microarray.

^‡^Genes determined by up or down regulation within the 1.5 fold change threshold.

^§^Cell adhesion genes: PR-D/S only (*Col1a1*, *Atp1b2*, *Ctnnd1*, *Rpsa*, and *Fat4*); PR-S only (*Cdh2*, *Edil3*, and *Astn1*).

^¶^DNA metabolism: PR-D/S only (*Tcf3*, *Mapk8*).

^ß^Stress response genes: C-D/S only (*Brsk1*, *Rarres2*); PR-D/S only (*Klk8*, *Myo6*, *Ndufa6*, *Col1a1*, *Mapk8*, and *Phlda3*).

**Table:** (b) Top 25 biological processes among the significantly regulated genes based on *P* values

GO ID	GO term	Frequency	Genome frequency	*P* value	Corrected *P* value
GO:0009987	Cellular process	0.68072	0.39346	2.0856*E* − 26	2.6196*E* − 23
GO:0071841	Cellular component organization or biogenesis at cellular level	0.27108	0.08456	8.0847*E* − 24	1.0154*E* − 20
GO:0071842	Cellular component organization at cellular level	0.25904	0.08100	1.2822*E* − 22	1.6105*E* − 19
GO:0071840	Cellular component organization or biogenesis	0.30120	0.10864	6.0965*E* − 22	7.6573*E* − 19
GO:0016043	Cellular component organization	0.28916	0.10476	7.5917*E* − 21	9.5351*E* − 18
GO:0008152	Metabolic process	0.50301	0.26565	1.9862*E* − 20	2.4947*E* − 17
GO:0044237	Cellular metabolic process	0.45181	0.22384	2.2263*E* − 20	2.7962*E* − 17
GO:0044238	Primary metabolic process	0.43976	0.22525	2.6340*E* − 18	3.3082*E* − 15
GO:0007399	Nervous system development	0.15060	0.04143	2.9821*E* − 15	3.7455*E* − 12
GO:0043170	Macromolecule metabolic process	0.37048	0.18900	5.7101*E* − 15	7.1719*E* − 12
GO:0051179	Localization	0.26807	0.11543	1.3853*E* − 14	1.7399*E* − 11
GO:0048699	Generation of neurons	0.11747	0.02708	1.8462*E* − 14	2.3188*E* − 11
GO:0030182	Neuron differentiation	0.11145	0.02443	1.9738*E* − 14	2.4790*E* − 11
GO:0044260	Cellular macromolecule metabolic process	0.33434	0.16512	2.9036*E* − 14	3.6469*E* − 11
GO:0022008	Neurogenesis	0.12048	0.02896	3.2387*E* − 14	4.0678*E* − 11
GO:0007275	Multicellular organismal development	0.25000	0.10479	3.3420*E* − 14	4.1975*E* − 11
GO:0006996	Organelle organization	0.16867	0.05448	4.7022*E* − 14	5.9060*E* − 11
GO:0048666	Neuron development	0.09639	0.01882	5.6471*E* − 14	7.0927*E* − 11
GO:0065007	Biological regulation	0.45783	0.27182	2.5651*E* − 13	3.2218*E* − 10
GO:0034641	Cellular nitrogen compound metabolic process	0.28614	0.13631	5.9844*E* − 13	7.5165*E* − 10
GO:0009058	Biosynthetic process	0.27108	0.12543	6.0854*E* − 13	7.6433*E* − 10
GO:0032502	Developmental process	0.25904	0.11705	6.6540*E* − 13	8.3574*E* − 10
GO:0006807	Nitrogen compound metabolic process	0.28916	0.13916	7.8812*E* − 13	9.8988*E* − 10
GO:0006139	Nucleobase, nucleoside, nucleotide, and nucleic acid metabolic process	0.27108	0.12602	7.9334*E* − 13	9.9643*E* − 10
GO:0031175	Neuron projection development	0.08434	0.01588	9.5266*E* − 13	1.1965*E* − 09

**Table 3:** Neurodevelopmental genes for genetic profiling based on hierarchical clustering analysis.

Gene symbol	Functional summary	Reference number	MGI ID	Fold change versus C				
				PR	C-D/S	PR-D/S	C-S	PR-S
*Sox11 *	Modulator of neocortical development	[4]	98359			↑3.84		
*Tcf3 *	Neural differentiation	[5]	1202876			↓1.62		
*Mapk8 *	Neuron projection development	[6]	1346861			↑1.50		
*Btbd6 *	Neuronal differentiation	[7]	3026623					↓1.96
*Mtap1b *	Axonal guidance and neuronal migration	[8]	1306778					↑3.83
*Eif2b5 *	Myelination and glial development	[9]	2446176					↓1.65
*Hmgcs1 *	Brain cholesterol pathways in myelination	[10]	107592					↑2.03
*Camk2n1 *	Neuronal development	[11]	1913509				↓1.81	↓1.87

## References

[1] de Boo HA, Harding JE (2006). The developmental origins of adult disease (Barker) hypothesis. *Australian and New Zealand Journal of Obstetrics and Gynaecology*.

[2] Velayo C, Ito T, Chisaka H, Yaegashi N, Okamura K, Kimura Y (2010). Effects of antenatal steroid therapy on neurodevelopment in an IUGR mouse model. *Fetal Diagnosis and Therapy*.

[3] Yoo SM, Choi JH, Lee SY, Yoo NC (2009). Applications of DNA microarray in disease diagnostics. *Journal of Microbiology and Biotechnology*.

[4] Li Y, Wang J, Zheng Y (2012). Sox11 modulates neocortical development by regulating the proliferation and neuronal differentiation of cortical intermediate precursors. *Acta Biochimica et Biophysica Sinica*.

[5] Ohtsuka T, Shimojo H, Matsunaga M (2011). Gene expression profiling of neural stem cells and identification of regulators of neural differentiation during cortical development. *Stem Cells*.

[6] Qu C, Li W, Shao Q (2013). c-Jun N-terminal kinase 1 (JNK1) is required for coordination of netrin signaling in axon guidance. *Journal of Biological Chemistry*.

[7] Sobieszczuk DF, Poliakov A, Xu Q, Wilkinson DG (2010). A feedback loop mediated by degradation of an inhibitor is required to initiate neuronal differentiation. *Genes and Development*.

[8] Utreras E, Jiménez-Mateos EM, Contreras-Vallejos E (2007). Microtubule-associated protein 1B interaction with tubulin tyrosine ligase contributes to the control of microtubule tyrosination. *Developmental Neuroscience*.

[9] Geva M, Cabilly Y, Assaf Y (2010). A mouse model for eukaryotic translation initiation factor 2B-leucodystrophy reveals abnormal development of brain white matter. *Brain*.

[10] Xiang Z, Valenza M, Cui L (2011). Peroxisome-proliferator-activated receptor gamma coactivator 1 *α* contributes to dysmyelination in experimental models of Huntington’s disease. *Journal of Neuroscience*.

[11] Ling K-H, Hewitt CA, Beissbarth T (2011). Spatiotemporal regulation of multiple overlapping sense and novel natural antisense transcripts at the nrgn and camk2n1 gene loci during mouse cerebral corticogenesis. *Cerebral Cortex*.

[12] Barker DJP (2007). The origins of the developmental origins theory. *Journal of Internal Medicine*.

[13] Tarry-Adkins JL, Chen J-H, Jones RH, Smith NH, Ozanne SE (2010). Poor maternal nutrition leads to alterations in oxidative stress, antioxidant defense capacity, and markers of fibrosis in rat islets: potential underlying mechanisms for development of the diabetic phenotype in later life. *FASEB Journal*.

[14] Boer K, Spliet WGM, van Rijen PC, Jansen FE, Aronica E (2010). Expression patterns of AMOG in developing human cortex and malformations of cortical development. *Epilepsy Research*.

[15] Fan Y, Zhu W, Yang M (2008). Del-1 gene transfer induces cerebral angiogenesis in mice. *Brain Research*.

[16] Wilson PM, Fryer RH, Fang Y, Hatten ME (2010). Astn2, a novel member of the astrotactin gene family, regulates the trafficking of ASTN1 during glial-guided neuronal migration. *Journal of Neuroscience*.

[17] Dembinska-Kiéc A, Polus A, Grzybowska J (2007). Nutritional factors and progenitor cell differentiation. *Genes and Nutrition*.

[18] Ishiuchi T, Misaki K, Yonemura S, Takeichi M, Tanoue T (2009). Mammalian Fat and Dachsous cadherins regulate apical membrane organization in the embryonic cerebral cortex. *Journal of Cell Biology*.

[19] Su H, Wang L, Huang W (2013). Immediate expression of Cdh2 is essential for efficient neural differentiation of mouse induced pluripotent stem cells. *Stem Cell Research*.

[20] de Kloet ER, Vreugdenhil E, Oitzl MS, Joëls M (1998). Brain corticosteroid receptor balance in health and disease. *Endocrine Reviews*.

[21] Barnes AP, Lilley BN, Pan YA (2007). LKB1 and SAD kinases define a pathway required for the polarization of cortical neurons. *Cell*.

[22] Garces MF, Sanchez E, Acosta BJ (2012). Expression and regulation of chemerin during rat pregnancy. *Placenta*.

[23] Yousef GM, Kishi T, Diamandis EP (2003). Role of kallikrein enzymes in the central nervous system. *Clinica Chimica Acta*.

[24] Lewis TL, Mao T, Arnold DB (2011). A role for Myosin VI in the localization of axonal proteins. *PLoS Biology*.

[25] Wirtz S, Schuelke M (2011). Region-specific expression of mitochondrial complex I genes during Murine Brain development. *PLoS ONE*.

[26] Paakinaho V, Makkonen H, Jääskeläinen T, Palvimo JJ (2010). Glucocorticoid receptor activates poised FKBP51 locus through long-distance interactions. *Molecular Endocrinology*.

[27] Ni L, Yang C-S, Gioeli D, Frierson H, Toft DO, Paschal BM (2010). FKBP51 promotes assembly of the Hsp90 chaperone complex and regulates androgen receptor signaling in prostate cancer cells. *Molecular and Cellular Biology*.

[28] Huo Y, Rangarajan P, Ling E-A, Dheen ST (2011). Dexamethasone inhibits the Nox-dependent ROS production via suppression of MKP-1-dependent MAPK pathways in activated microglia. *BMC Neuroscience*.

[29] Budziszewska B, Szymanska M, Leskiewicz M (2010). The decrease in JNK- and p38-MAP kinase activity is accompanied by the enhancement of PP2A phosphatase level in the brain of prenatally stressed rats. *Journal of Physiology and Pharmacology*.

[30] Sakoda H, Gotoh Y, Katagiri H (2003). Differing roles of Akt and serum- and glucocorticoid-regulated kinase in glucose metabolism, DNA synthesis, and oncogenic activity. *Journal of Biological Chemistry*.

[31] Zhao W, Qin W, Pan J, Wu Y, Bauman WA, Cardozo C (2009). Dependence of dexamethasone-induced Akt/FOXO1 signaling, upregulation of MAFbx, and protein catabolism upon the glucocorticoid receptor. *Biochemical and Biophysical Research Communications*.

[32] Szymańska M, Suska A, Budziszewska B (2009). Prenatal stress decreases glycogen synthase kinase-3 phosphorylation in the rat frontal cortex. *Pharmacological Reports*.

[33] Kapoor A, Petropoulos S, Matthews SG (2008). Fetal programming of hypothalamic-pituitary-adrenal (HPA) axis function and behavior by synthetic glucocorticoids. *Brain Research Reviews*.

[34] Cole MA, Kim PJ, Kalman BA, Spencer RL (2000). Dexamethasone suppression of corticosteroid secretion: evaluation of the site of action by receptor measures and functional studies. *Psychoneuroendocrinology*.

[35] Le PP, Friedman JR, Schug J (2005). Glucocorticoid receptor-dependent gene regulatory networks. *PLoS Genetics*.

